# Psychometric evaluation of the Chinese version of the revised American Pain Society Patient Outcome Questionnaire concerning pain management in Chinese orthopedic patients

**DOI:** 10.1371/journal.pone.0178268

**Published:** 2017-05-25

**Authors:** Huan Fang, Jingjuan Liang, Zhen Hong, Kenji Sugiyama, Takao Nozaki, Susumu Kobayashi, Tetsuro Sameshima, Hiroki Namba, Tetsuya Asakawa

**Affiliations:** 1 Department of Pharmacy, Jinshan Hospital of Fudan University, Shanghai, China; 2 Department of Orthopedics, Huashan Hospital of Fudan University, Shanghai, China; 3 Department of Neurology, Huashan Hospital of Fudan University, Shanghai, China; 4 Department of Neurosurgery, Hamamatsu University School of Medicine, Hamamatsu, Japan; Southern Illinois University Edwardsville, XX

## Abstract

The present study tested the clinical efficiency (item grouping, internal consistency of the subscales, construct validity, and clinical feasibility) of a widely used pain assessment system, the Mandarin version of the American Pain Society Patient Outcome Questionnaire (APS-POQ-R-C), in Chinese patients. We also attempted to investigate the current quality of pain management provided in orthopedic inpatient units in China and provide baseline data. First, we investigated the test–retest reliability of APS-POQ-R-C. In total, 236 orthopedic patients were evaluated. Our results showed that APS-POQ-R-C has satisfactory internal consistency and construct validity, although some items are not appropriate for orthopedic patients. Test–retest reliability outcomes indicated that APS-POQ-R-C is a satisfactory battery with acceptable validity and reliability, and is therefore recommended for pain management in future studies.

## Introduction

Pain is one of the most common symptoms associated with many disorders, is more frequent in surgical and cancer patients [1–5]. Unrelieved pain often causes serious negative consequences, including physiological and psychological impairments, which is harmful to patient outcome and quality of life [6]. Pain is, therefore, regarded as the fifth vital sign by many organizations such as Veterans Health Administration (VHA), National Pain Management Coordinating Committee, and American Pain Society (APS), etc. [7–9]. Elimination of pain, or at least reducing it to a tolerable level, in which pain management plays a crucial role, is emphasized by more and more clinicians [10–13].

Pain is a subjective experience encompassing multiple dimensions [14, 15]. In order to effectively manage pain, a quality improvement (QI) approach based on reliable and effective assessments is very important [16, 17]. A satisfactory assessment should include not only pain intensity (PI), but also all the domains affected by pain or affecting pain, such as ongoing pain assessment, interdisciplinary cooperation (nursing, clinical medicine, clinical pharmacy, and psychology), appropriate treatment, specialty care, and patient input [16]. Only an appropriate QI approach based on ideal pain assessments can provide a reliable evaluation of patients with pain, which contributes to effective pain management and is helpful for controlling pain. There are many measurements of pain, such as the Strategic and Clinical Quality Indicators in Postoperative Pain Management [18], Pain Treatment Satisfaction Scale [19], and the Patient Pain Questionnaire [20]. However, the most common assessment is the American Pain Society Patient Outcome Questionnaire (APS-POQ), which was developed in 1991 [21] and revised in 1995 and 2010 [1, 10, 14]. The latest version of APS-POQ was released in 2010. It is an investigator-reported measurement of six aspects of pain management: pain relief and severity; effect of pain on activity, sleep, and emotion; usefulness of information regarding pain treatment; nonpharmacological pain management; side effects of pain treatment; and participation in pain treatment decisions [10, 16]. This version has been translated into 11 languages [16]. Gordon et al. in 2010 investigated pain in 299 patients using an English version (APS-POQ-R). This study reported the initial psychometric properties of APS-POQ-R for QI, and the internal consistency of the instrument subscales and construct validity of APS-POQ-R were verified [16]. A later study evaluated the quality of an Icelandic version of APS-POQ-R (APS-POQ-R-I) in 143 patients. They found that APS-POQ-R-I was feasible, and the questionnaire had acceptable construct validity and reliability and is now recommended to evaluate the quality of pain management in hospitals in Iceland [10]. These studies were based on Caucasian populations; there are no data regarding the usage of APS-POQ-R among Mongolian populations. Many literatures suggested that sensitivity and tolerance of pain are different among ethnic groups. Tan et al compared the pain scores in the main four races in Singapore and found Indians having the highest mean pain score and using the highest amount of morphine [22]. Recently, Holmgaard et al also reported that people with dark eyes and hair exhibit higher pain sensitivity [23]. In this regard, it is meaningful to investigate the APS-POQ-R among Mongolian populations.

The aim of the present study is to test the item grouping, internal consistency of the instrument subscales, construct validity, and clinical feasibility of the Mandarin version of APS-POQ-R (APS-POQ-R-C) in Chinese orthopedic patients. We also investigated the relationships among items, subscales, and variables, which may potentially play a role in the prediction of outcomes affected by regional environments, cultural traditions, and native background behind the reported methods. We attempt to investigate the current quality of pain management provided in orthopedic inpatient units of China and provide baseline data. Moreover, we investigated the test–retest reliability of APS-POQ-R-C in the present study.

## Materials and methods

### Scale

The Mandarin version of APS-POQ-R was obtained directly from the American Pain Society website, which can be freely used without further permission. The questionnaire consists of 22 items, including pain severity (P1–P3), pain interference (P4, P5), adverse drug effects (P6), pain relief, participation in decision-making, satisfaction, and received pain treatment information. (P7–P10), nonpharmacological methods for treating pain (P11, P12), and whether patients received the help from investigators when completing the questionnaire (P13). Patients should read and sign informed consent at the beginning of the questionnaire.

During the preliminary investigation, we found it was difficult for the patients undergoing spine or hip joint surgery to complete the item of activities out of bed (P4b) because of the requirement of local or systemic immobilization in the first 24 h after surgery. Patients were usually confused by the major reason of disability, namely, by pain or by immobilization requirements. However, in order to retain the integrity of the scale, the item was not removed in subsequent tests. In this case, many subsequent attempts were made to make the patient understand the context of the question. Only 2 patients were usually confused and we excluded their responses and included them into blank forms.

A number of patients could not clearly distinguish the pain level (maximum or minimum), and may possibly have reported the same pain level. In this regard, two items (“pain relief,” P3 and “time spent in severe pain,” P7) were somewhat difficult for patients to report. We therefore supplemented the definition of “severe pain,” namely, a pain experience causing bad effect, according to a previous study [16].

### Patients

All patients were recruited into the cohort from the 50-bed ward of an orthopedics department of a 1216-bed comprehensive university hospital (February 2016 to June 2016). Patients of this department include fractures and acute musculoskeletal injury, spinal injury, wrist arthroscopy, hand and wrist fusions, shoulder and elbow replacements, hip disorders, and knee ligament reconstruction. The present study complied with the Declaration of Helsinki of the World Medical Association (2000), and was approved and supervised by the Ethics Committee of Huashan Hospital of Fudan University (approval number: 2015291). Informed consent was obtained from each patient or their relatives after all procedures were fully explained. The patients were recruited using the same methods as a previous study [16]. Briefly, a head nurse who was not directly involved in treating the patients was employed in the present study to select patients. The head nurse was well trained in performing assessments and data collection. The criteria for inclusion were age ≥14 years, orthopedic surgery in the past 72 h, pain in the past 24 h, conscious and responsive through the involvement process, and a native Chinese speaker without any communication obstacles. Once a patient was considered, the head nurse would communicate with him/her, and decided if the patient was included or not. If the patient met the inclusion criteria and agreed to participate in the study, the nurse then introduced all the items of the APS-POQ-R-C, and answered all questions from patients or their relatives personally. Patients were encouraged to complete the scale independently. Only those who underwent local or systemic immobilization had to complete the scoring with help of the head nurse. In this case, the nurse only objectively put down the patient’s answer regarding the scale. All the data were checked by the investigators of the present study.

### Statistical analysis

We used the same statistical analyzing methods as in a previous study [16]. Briefly, the difference in survey results between patients undergoing pharmacological therapy and without nonpharmacological therapy was tested by the Mann–Whitney U Test for skewed data. A correlation analysis was conducted to assess the relationships among items, subscales, and variables. Multiple stepwise linear regression analyses were used to determine and evaluate the effect of items, subscales, and variables on satisfaction outcomes and identify the predictors of satisfaction. Face validity was determined according to the methods of Shaik et al. [24]; 10 patients were involved in the preliminary experiment to acquire proper feedback. Kaiser–Meyer–Olkin (KMO) and the Barlett’s tests of sphericity were used to assess the appropriateness of using factor analysis on the data, and the principal component analysis with varimax rotation was used to confirm the construct validity of the questionnaire. For the evaluation of the original APS-POQ-R, scores of three items (P7, P8, P9, higher scores represent a favorable situation), were reversed to be in line with the other major items. Estimated items of P3 (pain relief) and P7 (time spent in severe pain) were normalized to 0–10 scales to match other items. The exploratory principal components factor analysis with varimax rotation was employed to extract components from all 18 continuous scales items (P1 to P9 listed in the APS-POQ-R). The internal consistency of APS-POQ-R-C was assessed with Cronbach’s alpha (α), which is acceptable if the α value is >0.7 [24]. Patients were selected randomly and administered the questionnaire twice before leaving the hospital, only 26 patients agreed to participate these tests. All these 26 patients were similar to the rest of the group. Since the satisfaction item was continuous, a linear regression was therefore employed to identify the satisfaction predictors. The correlation between scales and subscales of the two surveys was adopted to evaluate the test–retest reliability. All statistical analysis was completed with SPSS version 20.0 software (IBM SPSS Inc., Chicago, IL, USA,).

## Results

### Patient demographics

A total of 249 questionnaires were returned from 269 eligible participants (20 cases declined); of these, 236 were analyzed (13 were excluded because of returning blank forms, including 2 patients who were usually confused), for a response rate of 92.6%. The average completion time of the questionnaire was approximately 10 min (range, 6–20 min). The final participation rate was 87.7%.

Table 1 summarizes the characteristics of the participants (n = 236; 47.5% males and 52.5% females). Mean patient age was 54.8 ± 14.7 years (range, 16–82 years). Three categories of orthopedic surgery were involved in the present study: 47 upper limbs (19.9%), 113 lower limbs (47.9%) and 76 spinal columns (32.2%). With respect to education, 58 (24.6%) reported a college (or greater) level of education, and 144 (61.0%) reported a high school education.

**Table 1:** Demographic and clinical data of respondents.

Characteristic	Total Sample, N = 236 n (%) or Mean (S.D)
**Age**	54.8 (14.7)
**Gender**	
Female	124 (52.5)
Male	112 (47.5)
**Education**	
College or above	58 (24.6)
High school	144 (61.0)
Under high school	34 (14.4)
**Marital status**	
Married	214 (90.7)
Never married	22 (9.3)
**Surgery site**	
Upper limbs	47 (19.9)
Lower limbs	113 (47.9)
Spinal columns	76 (32.2)

### Initial survey of validity and reliability of APS-POQ-R-C

Regarding the initial component loading matrix of APS-POQ-R, the KMO was 0.80 while the probability value of Bartlett sphericity test was low (Bartlett test of sphericity: χ^2^ = 1229.8; p < 0.001); therefore, the indicated factor analysis was applicable. Six factors with Eigenvalues over 1 were identified, while the 67.57% total variance was explained.

According to this rotated component matrix, 18 items with different factor loading were affiliated to 6 groups, which were classified according to the criterion of loading coefficient >0.5. Except for “severity of itching” (P6c), which was isolated as a single factor, the initial results were similar to the subscales classified in original version, which were labeled with affective subscale (4 variables: P5a–P5d), pain severity and sleep interference subscale (5 variables: P1–P3, P4c, P4d), perceptions of care subscale (3 variables: P7–P9), activity interference subscale (2 variables: P4a, P4b), and adverse drug reaction (ADR) subscale (3 variables: P6a, P6b, and P6d).

We also investigated the Cronbach’s α and the corrected item-total correlation of total scale and subscales. The Cronbach’s α of initial total APS-POQ-R-C was 0.798, whereas the value based on standardized items was 0.820. The items were adjusted according to the results of accompanying changes of α value of total scale and subscales supervening after item deletion. Four items, namely, “activity interference out of bed” (P4b, 0.818), “severity of nausea” (P6a, 0.8), “severity of drowsiness” (P6b, 0.799), and “participation of pain treatment decision” (P8, 0.804), may increase the total α if deleted. After rounding, only deletion of P4b could enhance the α value. Spinal and hip joint surgery required immobilization after operation; thus, these patients could not get out of the bed, which was why some patients did not complete the interference section, in contrast to patients undergoing surgery on other parts of the body. In this regard, this item may not be appropriate for the patients strictly confined to bed.

Moreover, the loading coefficient of the itching item on the component of other 3 ADR items was 0.057, distinct from others 3, (severity of nausea 0.795, severity of dizziness 0.793, severity of drowsiness0.582); we attempted to retained the itching item in ADR subscales but it lowered α of ADR subscale to <0.6. The items of “activity interference out of bed” and “severity of itching” were therefore removed and the validity and reliability of the adjusted APS-POQ-R-C were recalculated.

### Construction of the final scale and subscale of APS-POQ-R-C for orthopedic surgery

Table 2 shows the component loading matrix of adjusted APS-POQ-R-C. After removing P4b and P6c, the KMO was 0.818 and P value of Bartlett test was <0.001. The exploratory factor analyses with 16 items produced 4 factors with Eigenvalue >1; namely, “pain severity and interference” (6 items: P1–P3, P4a, P4c, P4d), “affection” (4 items: P5a–P5d), “perceptions of care” (3 items: P7–P9), and “ADR” (3 items: P6a, P6b, P6d). The Eigenvalues after rotation were 3.47, 2.61, 1.80, and 1.73, respectively. The loading coefficients of items on each factor were >0.5, with the exception of the item of pain relief in the first hours, which had a loading coefficient >0.4 on 2 factors (“perceptions of care” and “pain severity and interference”). The pain severity and interference could explain 30.52% of variance, and all 4 factors could explain 60.07% of total variance.

**Table 2:** Component loading and rotated component matrix of adjusted APS-POQ-R-C.

	Component			
	Pain severity and interference subscale	Affection subscale	Perceptions of care subscale	Adverse drug reaction subscale
**P4c.** Pain interfered or prevented you from falling asleep	**.820**	.264	.003	.016
**P4d.** Pain interfered or prevented you from staying asleep	**.812**	.233	.028	.012
**P2.** Worst pain in 24 hours	**.743**	.233	.080	-.025
**P3.** Estimate of percentage of time in severe pain	**.703**	.192	.037	-.056
**P4a.** Pain interfered or prevented you from activities in bed	**.612**	-.030	.033	.084
**P1.** Least pain in 24 hours	**.503**	.214	.205	-.012
**P5c.** How much the pain caused you to feel frightened	.107	**.824**	.054	-.008
**P5d.** How much the pain caused you to feel helpless	.206	**.813**	.049	.054
**P5b.** How much the pain caused you to feel depressed	.238	**.721**	.066	.143
**P5a.** How much the pain caused you to feel anxious	.355	**.666**	.248	.066
**P8.** Were you allowed to participate in decisions about pain treatment	-.103	.131	**.834**	-.021
**P9.** How satisfied are you with the results of your pain treatment	.162	.106	**.826**	.079
**P7.** Pain relief in the first 24 hours (%)	.430	.025	**.544**	.192
**P6d.** Severity of dizziness	.031	.111	.063	**.794**
**P6a.** Severity of nausea	-.055	.011	.011	**.768**
**P6b.** Severity of drowsiness	.047	.043	.058	**.653**

Kaiser-Meyer-Olkin measure of sampling adequacy: 0.818.

Bartlett test of sphericity: *X*^*2*^ = 1278.2; p < 0.001.

Table 3 shows the correlations of all items, including the subscales. All of the correlations were >0.3, which indicated each item was consistent with the measurement behavior of the subscale and could not be discarded [25]. The Cronbach’s α of total scale was 0.818 and the standardized value was 0.83; these data indicated that the reliability of total APS-POQ-R-C of the final version was good. α of 4 subscales were 0.819 (“pain severity and interference”), 0.812 (“affection”), 0.609 (“adverse reactions”) and 0.618 (“perceptions of care”) (Table 3). Although, as in the α of “ADRs” and “perceptions of care,” 0.7 is usually regarded as an acceptable internal consistency; however, for the fewer and important items, thresholds can be adjusted to 0.6 [16, 26].

**Table 3:** Final subscale item-total statistics.

	Subscale Mean if Item Deleted	Subscale Variance if Item Deleted	Subscale Corrected Item-Total Correlation	Subscale Cronbach's Alpha if Item Deleted
**Pain severity and interference subscale (α = .819)**				
**P1.** Least pain in 24 hours	12.1717	96.470	.442	.824
**P2.** Worst pain in 24 hours	9.8197	81.002	.648	.782
**P3.** Estimate of percentage of time in severe pain	11.2232	77.821	.590	.789
**P4a.** Pain interfered or prevented you from activities in bed	10.1073	80.269	.411	.833
**P4c.**Pain interfered or prevented you from falling asleep	11.0601	64.272	.770	.744
**P4d.**Pain interfered or prevented you from staying asleep	11.3691	64.751	.760	.747
**Affection subscale (α = .812)**				
**P5a.**How much the pain caused you to feel anxious	1.3750	11.378	.623	.791
**P5b.** How much the pain caused you to feel depressed	1.7586	14.452	.625	.767
**P5c.** How much the pain caused you to feel frightened	1.9526	15.266	.642	.766
**P5d.** How much the pain caused you to feel helpless	1.8966	13.946	.697	.736
**Adverse drug reaction Subscale (α = .609)**				
**P6a.** Severity of nausea	1.2103	4.719	.424	.501
**P6b.** Severity of drowsiness	1.1545	5.769	.342	.609
**P6d.** Severity of dizziness	1.0601	4.807	.493	.397
**Perceptions of care subscale (α = .618)**				
**P7.** Pain relief in the first 24 hours (%)	2.4638	17.122	.366	.652
**P8.** Were you allowed to participate in decisions about pain treatment	4.3489	19.245	.412	.541
**P9.** How satisfied are you with the results of your pain treatment	4.6766	22.416	.583	.399

Cronbach's α of Total Scale: 0.818

Table 4 shows the correlation matrix among subscales. The results indicated that the pairwise correlation among 3 subscales, including “pain severity and interference,” “affection” and” perceptions of care,” were higher than that between the ADR subscale and others. The highest pairwise correlation coefficient was between “pain severity and interference” and “affection” (0.508), which indicated good independence of the 4 subscales (Table 4).

**Table 4:** Correlation matrix among subscales.

	Pain severity and interference	Affection	ADR	Perceptions of care
**Pain severity and interference**	1.000	.508^**^	.095	.488^**^
**Affection**	.508^**^	1.000	.213^**^	.423^**^
**ADR**	.095	.213^**^	1.000	.164^*^
**Perceptions of care**	.488^**^	.423^**^	.164^*^	1.000

*p<0.05

**p<0.01

As to the test–retest reliability of the final APS-POQ-R-C (obtained from 26 patients). The correlation coefficient of the subscales and total scales between two tests of final APS-POQ-R-C were extremely high (0.714–0.914). As for the independent sample test, no significant difference was found among the 4 subscales and total scales between two tests. The intraclass correlation coefficient (ICC) were >0.80 for total scale, and >0.75 for all subscales, except that the perception of care subscale was 0.654. According to these results, the test–retest reliability of the final APS-POO-R-C was verified.

### Construction of the model of satisfaction prediction

Tests of independent samples were employed to assess the differences of APS-POQ-R-C survey results between patients undergoing nonpharmacological therapy and those without nonpharmacological therapy. Of the 16 items in the scale, only “the level of percentage of time spent in severe pain” and “pain interference with falling asleep” (P3, P4c) were significantly higher in patients undergoing nonpharmacological therapy. No difference was found in the “patient satisfaction,” “participation in decision-making” and other items (Table 5).

**Table 5:** Differences of APS-POQ-R-C between patients with nonpharmacological therapy and nonusers.

	Non-medicine Methods to Relieve Pain	N	Mean	Std. Deviation	Mann Whitney U Test
Least pain in 24 hours	YES	142	.9859	1.19090	.796
	NO	94	.9574	1.04640	
Worst pain in 24 hours	YES	142	3.5211	2.09937	.107
	NO	94	3.0000	1.72894	
Estimate of percentage of time in Severe pain	YES	142	2.2465	2.49876	.013
	NO	94	1.4681	2.06716	
Pain interfered or prevented you from activities in bed	YES	142	3.2606	2.78211	.090
	NO	94	2.6596	2.57178	
Pain interfered or prevented you from falling asleep	YES	142	2.4437	3.12964	.040
	NO	94	1.5957	2.39767	
Pain interfered or prevented you from staying asleep	YES	141	2.0851	3.10642	.081
	NO	92	1.3152	2.32487	
How much the pain caused you to feel anxious	YES	142	1.0000	2.05250	.865
	NO	91	.9011	1.66035	
How much the pain caused you to feel depressed	YES	142	.5493	1.41711	.583
	NO	91	.5934	1.35792	
How much the pain caused you to feel frightened	YES	142	.3099	1.17409	.202
	NO	91	.5055	1.35297	
How much the pain caused you to feel helpless	YES	142	.3944	1.37329	.456
	NO	91	.5495	1.50010	
Severity of nausea	YES	142	.5704	1.60406	.673
	NO	92	.4130	1.24169	
Severity of drowsiness	YES	141	.6312	1.40108	.324
	NO	92	.4457	1.12283	
Severity of dizziness	YES	142	.6972	1.47311	.791
	NO	92	.5870	1.15931	
Pain relief in the first 24 hours (%)	YES	141	6.7305	3.09349	.384
	NO	91	7.0110	3.10016	
Were you allowed to participate in decisions about pain treatment	YES	141	8.8227	2.63841	.098
	NO	89	8.7416	2.27400	
How satisfied are you with the results of your pain treatment	YES	142	9.1690	1.39387	.767
	NO	90	8.9444	1.81356	

Table 6 shows the regression model of satisfaction predictors. To screen the patient satisfaction predictors, satisfaction item (P9) was employed as the dependent variable, while other items in the final APS-POQ-R-C (age, binary variables including sex, information received about pain treatment [P10], and nonpharmacological methods to relieve pain [P11]) were selected as independent variables. The model contained 5 variables: “pain relief,” “participation in pain treatment decisions,” “activity interference in bed,” “depression caused by pain,” and “least pain in 24 hours” (adjusted R^2^ = 0.29, F = 20.36, p < 0.001). Our data indicated that these five items may be important components that influence pain satisfaction in patients (Table 6).

**Table 6:** Regression model of satisfaction predictors.

	Unstandardized Coefficients		Standardized Coefficients	t	Sig.
	Beta	SE	Beta		
**Constant**	7.305	.389		18.755	.000
**Pain relief in the first 24 hours (%)**	.107	.030	.210	3.535	.000
**Were you allowed to participate in decisions about pain treatment**	.186	.035	.294	5.230	.000
**Pain interfered or prevented you from activities in bed**	-.101	.034	-.176	-3.010	.003
**How much the pain caused you to feel depressed**	-.166	.065	-.147	-2.554	.011
**Least pain in 24 hours**	-.190	.079	-.139	-2.400	.017

### Descriptive statistics of final version of APS-POQ-R-C items

As in the original version of APS-POQ-R-C, the continuous scales as well as the necessary additional items for pain treatment were included in the final version scale. Table 7 shows the numeric rating scales (NRS) data measured from 0–10. The mean of “worst pain in 24 hours” was 3.3136 ± 1.97301 (indicating mild degree of pain), “pain relief” was 68.405% ± 30.9244% and “satisfaction” was 9.0819 ± 1.57007. These data showed that the scores of the “degree of satisfaction” were high, which indicated that pain in these postoperative patients was generally under control.

**Table 7:** Results of APS-POQ-R-C.

Continuous scales	N	Minimum	Maximum	Mean	SD
Least pain in 24 hours	236	0.00	6.00	.9746	1.13334
Worst pain in 24 hours	236	0.00	10.00	3.3136	1.97301
Estimate of percentage of time in severe pain (%)	236	0.00	100.0	19.364	23.6287
Pain interfered or prevented you from activities in bed	236	0.00	10.00	3.0212	2.71081
Pain interfered or prevented you from falling asleep	236	0.00	10.00	2.1059	2.88529
Pain interfered or prevented you from staying asleep	233	0.00	10.00	1.7811	2.84350
How much the pain caused you to feel anxious	233	0.00	10.00	.9614	1.90581
How much the pain caused you to feel depressed	233	0.00	7.00	.5665	1.39151
How much the pain caused you to feel frightened	233	0.00	10.00	.3863	1.24782
How much the pain caused you to feel helpless	233	0.00	10.00	.4549	1.42299
Severity of nausea	234	0.00	10.00	.5085	1.47145
Severity of drowsiness	233	0.00	7.00	.5579	1.29898
Severity of dizziness	234	0.00	8.00	.6538	1.35685
Pain relief in the first 24 hours (%)	232	0.00	100.00	68.405	30.9244
Were you allowed to participate in decisions about pain treatment	230	0.00	10.00	8.7913	2.49889
How satisfied are you with the results of your pain treatment	232	2.00	10.00	9.0819	1.57007

**Fig 1:**
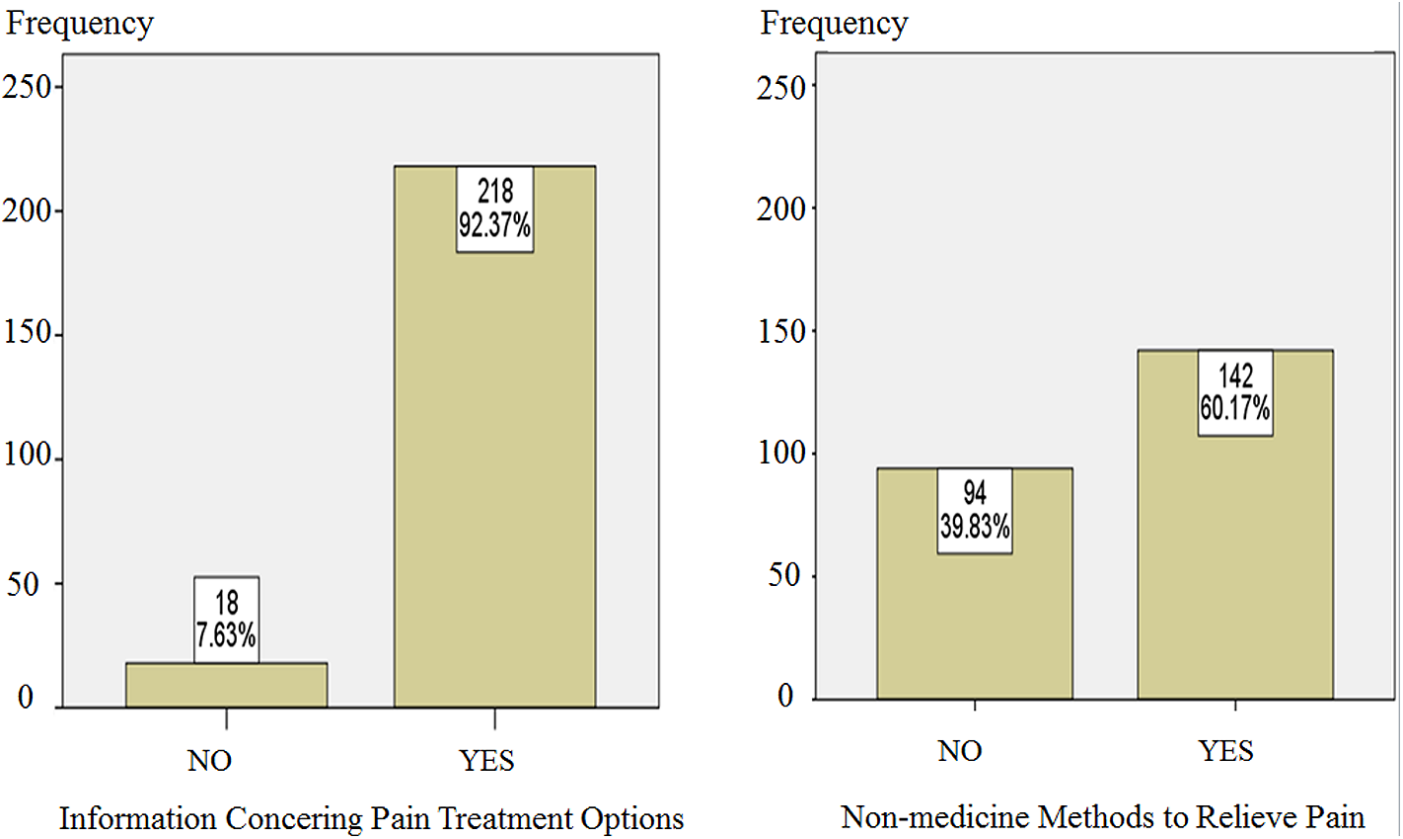
Distribution of received information and treatment selection in the enrolled patients. There are 92.37% of the patients received information about pain treatment options, and 60.17% of patients selected non-pharmacological methods to relieve pain.

With regard to additional items for pain, we calculated and summarized the data using the methods by Gordon et al. [16] and Zoega et al. [10]. Fig 1 shows that 92.37% of the patients received information about pain treatment options, and 60.17% of patients selected non-pharmacological methods to relieve pain. Table 8 shows that the average “usefulness of information received for pain treatment” was 8.7477 ± 2.08475. As for the level of how often the clinician encouraged nonpharmacological methods, most of the patients (66.2%) considered that the clinician “sometimes” encouraged the nonmedication methods, while “18.2%” selected “often” and 15.6% selected “never”

**Table 8:** Usefulness of received information and frequency of nonmedication methods encouragement.

	N	Minimum	Maximum	Mean	SD
**Usefulness of information for pain treatment**	218	0.00	10.00	8.7477	2.08475
**How often a doctor or nurse encourage nonmedication methods (N = 231)**	Frequency	Valid Percent		Cumulative Percent	
**1.00 (never)**	36	15.6		15.6	
**2.00 (sometimes)**	153	66.2		81.8	
**3.00(often)**	42	18.2		100.0	

## Discussion

### Evaluating the reliability of the total scale

Although the use of APS-POQ (1995) and APS-POQ-R (2010) is well documented [10, 16, 17, 27], there are no studies that evaluate these scales in Asian populations. In the present study, indeed we found that APS-POQ-R-C has a satisfactory internal consistency for total scale (original version α = 0.798, and final version α = 0.818) in Chinese patients. In the previous studies using APS-POQ-R in different ethnic groups, the internal consistency was quite different among different populations. The Cronbach α in Gordon’s study was 0.86 [16], and was 0.84 in final Iceland version [10], however, low in Danish (Cronbach α = 0.54) and Australian (Cronbach α = 0.63) cohorts [28]. The difference may derive from different sensitivity and tolerance of pain among different populations, different pain management system, different prescribing habits against pain, and different understanding of the items caused by the culture difference. Based on these results, we suggest that APS-POQ-R-C is a valid and reliable battery for pain evaluation in Chinese patients, although some revision (removal of p4b and p6c) is recommended in the actual practice for orthopedic patients. To the best of our knowledge, the present study is the first report to perform APS-POQ-R among Asian patients.

### Modifying the items of the APS-POQ-R-C

Moreover, several differences were found in comparison with the previously investigated American original ASP-POQ-R [16] and the Icelandic version [10]. First, we had to delete“itching item” (P6c) subscales because adding the “itching” item into the ADR subscale may lower the Cronbach’s α of subscale to <0.6, while deleting this item may enhance the Cronbach’s α to >0.6. Generally, ADR symptoms can be divided into type A or type B [29, 30]. In this study, the “itching” item was type B, whereas the others were type A. For patients with pain, opioid intake is an important factor that should be seriously taken into account in clinical practice, since release of histamine by opiates is a potential factor causing allergic reaction. In China, nonsteroidal anti-inflammatory drugs, instead of opioids, are the most widely used drugs to treat pain after orthopedic surgery. The present study evaluated orthopedic patients, whose opioid analgesics usage were lower than that of the tumor patients (related to less itching) in the previous study [16]. Differences of drug administration caused by patient selection between the present study and previous ones may be an explanation concerning the difference of the “itching” item. Second, we deleted the item of “interference with activities out of bed” (P4b) and then merged the “pain severity and sleep interference” subscale and the “activity interference” subscale into one subscale in the final APS-POQ-R-C according to the results of the exploratory factor analysis. The removal of P4b enhanced the Cronbach’s α of total scale to >0.8. For some orthopedics patients, immobilization was required during the first postoperative 24 h. Also, it was difficult for some patients (such as those who received spinal or hip joint surgery) to distinguish whether the disability was caused by pain, which might cause confusion or difficulty to complete P4b, consequently lowering the Cronbach’s α. After removal of P4b, the remaining item of “activity interference subscale” (P4a) was therefore classified in the “pain severity and sleep interference” subscale.

### The lower Cronbach’s α of “ADR” and “perceptions of care” subscales in the present study

We also found that the Cronbach’s α of the ADR subscale in the present study (0.609) was slight lower than that in the American study (0.609 vs. 0.63) and the Icelandic study (0.609 vs. 0.75 respectively) [10, 16]. One reason is the items of the present study were fewer (3 vs. 4 in the American study). Moreover, as in the American study, the ADRs of the orthopedic patients in the present study were also very low; this might be another explanation for the low Cronbach’s α in the present study. Another problem in the present study was the Cronbach’s α of “perceptions of care” subscale was only 0.618. This result was not as satisfactory as that in “total scale” and “affection” subscales. Similar to the ADR subscales, too few items may lower the Cronbach’s α. Although all items of the “perceptions of care” subscale (P7, P8, and P9) were well described, investigation of “satisfaction and participation” may be confused with the investigation to “medical or nursing quality” by some patients in the pain treatment questionnaire. A previous study reported that patients were satisfied with pain management even with an uncontrolled pain level [27]. In addition, the Cronbach’s α of the “satisfaction” subscale was low in Dihle’s study [1], which investigated the reliability and validity of an older version of APS in orthopedic postoperative patients; therefore, further study was needed to investigate non-surgical patients and other surgery patients.

### Comparing the scores between patients with and without nonpharmacological therapy

With regard to the results comparing the difference between patients undergoing nonpharmacological therapy and those without nonpharmacological therapy, only the scores of 2 items, namely “percentage of time spent in severe pain” along with “pain interference with falling asleep” were significantly higher in the nonpharmacological group. There was no difference for the “degree of the satisfaction and participation” between the two groups. Different from the American version, patients in the nonpharmacological methods group had significantly lower satisfaction scores and significantly higher “worst pain” scores.

### Factors affecting the satisfaction of pain treatment

Along with the items of “pain relief” (P7) and “level of participation” (P8) in the American version, other predictors, namely “least pain” (P1), “depression” (P5b), and “activity interference in bed” (P4a) were also included in the APS-POQ-R-C; however, “the time in severe pain” (P3) and “information received” (P10) in the American version were not included. When the subscales scores were selected as the independent items, only the items of “pain severity and interference” subscale and “affective subscale” demonstrated a significant contribution to the changes of “satisfaction” or “perception of care” subscale, while the ADR subscales did not influence the result.

### The test–retest reliability of the APS-POQ-R-C

The test–retest reliability of the APS-POQ was first verified in the present study. Spearman correlation and ICC are usually employed as indicators of reliability coefficient, while the ICC is more rigorous. Both indexes were adopted in this study to test the test–retest reliability. Test–retest reliabilities for each of the 4 subscales along with the total scale were statistically significant (p < 0.001). Except for the ICC of “perception of care” subscale of 0.654, which was acceptable and might be caused by some patients confusing the items of “satisfaction and participation concerning pain care” with the “investigation of medical or nursing quality,” other subscales showed very satisfactory retest reliability coefficients (0.769–0.920). Good test–retest reliability of the first three factors (pain severity and interference, affection, and ADR) indicated a minimal measurement error related to random variance [31]. Our test–retest reliability results confirmed the stability of APS-POQ-R-C. The second scoring process during the test–retest reliability may be influenced by the feeling about the medical service including doctor, nurse or hospital.

## Conclusions

Taken together, the present study first investigated APS-POQ-R-C among Chinese orthopedic patients. However, we selected different patients from the previous studies in different languages, and it is difficult to draw a conclusion by simply comparing the present study with the previous ones. Our data indicate that APS-POQ-R-C has satisfactory internal consistency and construct validity in general. Moreover, the results of the test–retest further indicated that APS-POQ-R-C is valid and reliable, and is therefore recommended for pain management in Chinese orthopedic patients.
